# Effect of Time and Temperature on the Detection of PRRSV RNA and Endogenous Internal Sample Control in Porcine Tongue Fluids †

**DOI:** 10.3390/vetsci12010059

**Published:** 2025-01-15

**Authors:** Isadora F. Machado, Onyekachukwu H. Osemeke, Kent Doolittle, Cesar A. A. Moura, Lucina Galina Pantoja, Giovani Trevisan, Phillip Gauger, Daniel C. L. Linhares

**Affiliations:** 1 Department of Veterinary Diagnostic and Production Animal Medicine, College of Veterinary Medicine, Iowa State University, Ames, IA 50011, USA; imachado@iastate.edu (I.F.M.);; 2 IDEXX Laboratories Inc., Westbrook, ME 04092, USA; 3 Iowa Select Farms, Iowa Falls, IA 50126, USA; 4 Genus plc PIC, Hendersonville, TN 37075, USA

**Keywords:** PRRSV, RT-qPCR, internal sample control, swine, tongue fluids, disease monitoring

## Abstract

Population-based sampling has improved pathogen monitoring in the US swine industry by increasing sensitivity while reducing costs. Postmortem tongue fluids (TF) have emerged as a practical option for monitoring porcine reproductive and respiratory syndrome virus (PRRSV) in breeding herds, but limited data exist on optimal storage conditions. This study evaluated PRRSV RNA detection via RT-qPCR in TF samples under various storage times, temperatures, and viral loads. Additionally, the porcine endogenous reference gene (internal sample control, ISC) was assessed for monitoring sample quality. Three studies were conducted: Study 1 compared fresh TF samples to freeze–thaw TF samples, with significantly lower cycle threshold (Ct) values in fresh samples. Studies 2 and 3 evaluated PRRSV RNA stability across six time points (0 to 336 h) and four temperatures (−20 °C, 4 °C, 22 °C, and 34 °C). At 4 °C, Ct value variation was minimal (≤1.1) over 14 days (336 h), with no significant changes within 48 h. In contrast, storage at 22 °C and 34 °C resulted in increased Ct values. The ISC RNA showed similar stability patterns, maintaining quality at 4 °C but degrading at higher temperatures. In conclusion, storing TF at ≤4 °C for up to 14 days for PRRSV and ISC RNA detection is the best scenario, ensuring optimal diagnostic quality.

## Simple Summary

Monitoring pathogens is essential to prevent, control and eliminate pathogens in the swine production systems. Postmortem tongue fluids collected from dead pigs are a practical method to assess the circulation of porcine reproductive and respiratory syndrome virus (PRRSV), a well-known pathogen to the swine industry that causes significant financial losses. However, there is limited information on optimal storage conditions to ensure correct test results. This study assessed PRRSV genetic material in tongue fluid samples stored under different practical field conditions (temperatures and time periods). Keeping the samples cold at fridge temperatures (4 °C) for up to 14 days was the best scenario to maintain optimal diagnostic quality. Storing samples at warmer temperatures, such as room temperature or higher (22 °C or 34 °C), negatively affected testing results. With these findings, producers, veterinarians, and diagnosticians can better handle the samples and correctly interpret the results. This will improve efforts to control and prevent diseases in swine, contributing to healthier herds.

## 1. Introduction

Over the past decade, population-based sampling for pathogen monitoring has expanded within the United States swine industry [1]. This broader sampling approach enhances herd sensitivity without increasing cost, time, and labor compared to bleeding a subset of animals. Common examples of population-based samples include oral fluids, processing fluids, udder wipes, and family oral fluids [2,3,4,5,6]. Likewise, post-mortem tongue fluids (TF) have emerged as a viable option for porcine reproductive and respiratory syndrome virus (PRRSV), Influenza-A virus (unpublished), and porcine circovirus type 2 monitoring in breeding herds [7,8,9,10].

Porcine reproductive and respiratory syndrome (PRRS) virus, in particular, continues to cause significant financial losses to the swine industry—estimated at USD 664 million annually in the United States and EUR 126 per sow in Dutch sow herds [10,11,12]. These economic impacts have driven efforts to improve monitoring programs, including investigating recent sampling methods, such as TF.

The composition of the TF specimen is complex, consisting of blood, saliva, respiratory mucus, and environmental contaminants. Also, amniotic fluid and meconium may be observed depending on the age of the sampled animal, such as stillborns [7,9]. Furthermore, since pigs are naturally curious animals, they use their mouths to interact with surrounding objects, potentially increasing the chances of finding pathogens present in the environment when TF is obtained [13,14,15]. However, saliva contains degradative enzymes, bacterial proteases, and ribonucleases, which could affect the stability of nucleic acids, which are often targets of quantitative polymerase chain reaction (qPCR) [16,17,18].

There is considerable variability in TF collection, handling, and processing methods. Identifying optimal storage conditions maintains the diagnostic quality of the sample, further aiding the effectiveness of a PRRSV monitoring program. Moreover, diagnostic testing can be trustworthy by concomitantly detecting the porcine endogenous reference gene (internal sample control [ISC]), as it is used to assess sample quality in PCR-based assays [19,20,21,22]. Hence, it is crucial to determine the best practices for TF handling in farms and diagnostic laboratories. Thus, the hypothesis is that PRRSV and ISC RNA detection in TF are affected by storage conditions due to potential genetic material deterioration at high temperatures and over extended storage periods. This study aimed to determine the time and temperature effects on PRRSV and ISC RNA detection in TF under common scenarios experienced by individuals collecting, storing, and submitting samples for molecular testing.

## 2. Materials and Methods

### 2.1. Experimental Design

**Figure 1:**
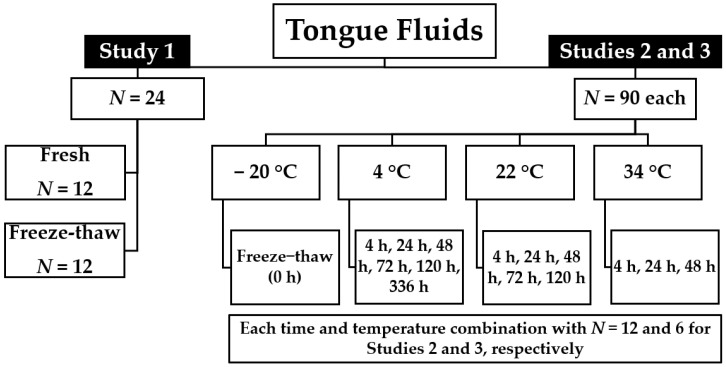
Process chart on the detection of porcine reproductive and respiratory syndrome virus and internal sample control RNA in tongue fluid specimen across studies 1, 2, and 3. N indicates the number of tongue fluids aliquots per study. Study 1: Fresh and freeze–thaw effect comparison. Study 2: Time and temperature effect with a high viral load sample. Study 3: Time and temperature effect with a low viral load sample.

Three studies were conducted to characterize the effect of time and temperature on PRRSV and ISC RNA detection by RT-qPCR in TF (Figure 1). In Study 1, the effect of a single freeze–thaw cycle on PRRSV RT-qPCR Ct values in a TF was assessed: 12 fresh TF replicates and 12 freeze–thaw TF replicates were tested. In Study 2, PRRSV and ISC RNA RT-qPCR detection was assessed at 6-time intervals (0 h, 24 h, 48 h, 72 h, 120 h, and 336 h) and 4 temperatures (−20 °C, 4 °C, 22 °C, and 34 °C), with combinations of time and temperature producing 15 treatments, repeated in 6 replicates and tested in two PCR plates in duplicates, i.e., two extraction and amplification protocols for each of the 15 treatments. Study 3, similar to the protocol described in Study 2, was executed with a low viral load TF. Freeze–thaw treatment (−20 °C at hour 0) served as the baseline for comparison for Studies 2 and 3. General linear and mixed-effect regression models were used for the data analyses to evaluate the Cycle threshold (Ct) value difference between treatments.

This study was approved by the Institutional Biosafety Committee (IBC) of Iowa State University, IA, USA, under protocol IBC-24-096 “Field surveillance of swine pathogens using post-mortem tissues” and by the Institutional Animal Care and Use Committee (IACUC) of Iowa State University, IA, USA, under protocol IACUC-22-101 “Field surveillance for swine pathogens”.

### 2.2. Sample Collection

Two 7000 sows breed-to-wean PRRSV-unstable [23] herds (A and B) that broke with PRRSV RFLP 1-4-4 L1C.5 in the U.S. Midwest were selected for sampling. From each herd, on two different sampling days (herd A on the first day and B on the second), 500 tongue tips (*n* = 250 per herd) were collected from stillborn and dead piglets (0–21 days old) found within 24 h before the farm visit, representing the pre-weaning mortality in the breeding herd. Thus, the number of samples depended on the availability of dead piglets on the sampling day.

The TF from Studies 1 and 2 were collected from a herd (A) with reported processing fluid (PF) PRRSV Ct values consistently below 20; thus, we defined the TF sample as “high viral load”. In contrast, Study 3 samples were collected from a herd (B) with reported PF PRRSV Ct values consistently above 30, defining the TF samples as “low viral load”. Thus, viral loads were defined based on quantitative PRRSV PCR Ct values, where lower Ct values indicate higher concentrations of viral RNA, and higher Ct values indicate lower concentrations of viral RNA.

To sample each dead piglet, forceps were used to hold the tongue in place for approximately three centimeters of the tissue to be severed with scissors and placed into a disposable plastic bag, as elsewhere described [7,8]. Following the sampling, the bags with the tongues (*n* = 250 tongues/bag) were refrigerated at 4 °C for three hours, i.e., until arriving at the laboratory.

### 2.3. Specimen Handling

In Studies 1 and 2, 110 mL of TF was extracted from the bags using a 50 mL syringe and placed into three 50 mL sterile polypropylene tubes (Falcon^®^, Mexico City, Mexico). The tubes containing the TF were centrifuged at 1400× *g* for 5 min, and the supernatant (80 mL) was transferred into a sterile 250 mL glass beaker recipient. Afterward, 0.7 mL of the supernatant was aliquoted into each of 114 5-mL round-bottom polystyrene tubes (Falcon^®^), which were allocated as follows: 24 aliquots for study 1 (12 stored at 4 °C for fresh treatment and 12 at −20 °C for freeze–thaw treatment) and 90 aliquots for Study 2, stored at −20 °C, following its study design (Figure 1). In Study 3, a different TF sample was used, but the same process described in Study 2 was followed, resulting in 60 mL of supernatant TF being aliquoted in 90 aliquots, each with 0.6 mL. For the thawing process, the temperature at 4 °C for 6 h was chosen based on previous literature available for better integrity of PRRSV RNA within the samples [24].

#### 2.3.1. Study 1

Study 1 was conducted to evaluate the effect of freeze–thaw on PRRSV RNA RT-qPCR detection in TF (Figure 1). On day 1, after the aliquoting process of the high viral load sample, 12 aliquots were promptly tested for PRRSV RNA by RT-qPCR, i.e., five hours after the farm sampling (fresh treatment). The other 12 aliquots were frozen at −20 °C for 18 h (freeze–thaw treatment), followed by a thawing process, and tested for PRRSV RNA by RT-qPCR on day 2.

#### 2.3.2. Study 2

All aliquots from the high viral load TF sample were frozen at −20 °C on day 1, aiming to have all samples tested by day 15. Thus, all treatments underwent a freeze–thaw cycle. Following the time and temperature study design (Figure 1), the samples were thawed and placed into their designed treatment [25].

The time–temperature combinations were selected based on questions from veterinarians and producers submitting TF for testing. Briefly, the −20 °C treatment captures the situation of freezing TF bags for future pooled testing, such as samples from several rooms or days. The 4 °C represents the farm refrigerator storing samples for various periods before testing. The 22 °C treatment represents room temperature, and 34 °C represents a hypothetical situation when samples are exposed to elevated temperatures. The −20 °C and 4 °C temperatures were attained using a refrigerator, and the 22 °C and 34 °C treatments were conducted using a water bath. The treatments’ temperatures were monitored daily, with no uncommon variations observed.

On day 15, RNA detection assays were conducted using two 96-well PCR plates. Each PCR plate accommodated 15 treatments, with 6 replicates per treatment, resulting in a total of 94 wells utilized in each plate, taking into consideration the amplification (*n* = 2) and extraction (*n* = 2) controls. The treatments were tested in duplicates, positioned in a similar arrangement across the two PCR plates.

#### 2.3.3. Study 3

In Study 3, the same protocol executed in Study 2 was followed, except it was performed with a low viral load TF sample and without a duplicate PCR plate.

### 2.4. RNA Extraction and PRRSV RT-qPCR Assay

Extraction of RNA from TF was carried out following the manufacturer’s instructions (RealPCR*DNA/RNA Magnetic Bead Kit; IDEXX Laboratories, Inc., Westbrook, ME, USA). An automated extraction equipment was used in the process (Kingfisher Flex System Magnetic Beads Processor, Thermo-Fisher Scientific, Waltham, MA, USA). Briefly, the lysis working solution was prepared with lysis buffer (176 µL), carrier RNA (4 µL), and proteinase K (20 µL) per sample and placed into a 96-well deep well plate. Thereafter, samples (200 µL) were individually incubated in the wells containing the lysis working solution for 15 min. The magnetic beads were retrieved and washed for 3 min with 600 µL each of Wash Solution I, Wash Solution II, and 80% ethanol, followed by a 10-min drying period. Finally, nucleic acids were eluted from the magnetic beads with the elution buffer solution (100 µL) over a 5-min period.

The PRRSV RT-qPCR assays were performed as instructed by the manufacturer (RealPCR* PRRSV-1 and PRRSV-2 Multiplex RNA Mix and Master Mix; IDEXX Laboratories, Inc., Westbrook, ME, USA). Briefly, extracted nucleic acids (5 µL) were transferred to PCR plate wells, each containing the Master mix (10 µL) and multiplex RNA mix (10 µL) containing primers and probes for both PRRSV and ISC. Thereafter, plates were placed onto a thermal cycler (7500 Fast Real-Time PCR System, Applied Biosystems, Foster City, CA, USA), using the following cycling conditions with standard ramp rate instructed by the manufacturer: one reverse transcription cycle at 50 °C for 15 min, one denaturation cycle at 95 °C for 1 min, and 45 amplification cycles (each having a set at 95 °C for 15 s and 60 °C for 30 s). Amplification data were analyzed using the ‘auto baseline’ with the Ct value manually adjusted to 10% of the peak reading of the positive amplification control. Samples with Ct values < 40 were considered positive per the manufacturer’s recommendation. Quality controls were included in each RT-qPCR plate, consisting of a positive (IDEXX Laboratories, Inc., Westbrook, ME, USA) and a negative (PCR grade water; IDEXX Laboratories, Inc., Westbrook, ME, USA) amplification controls and a positive and a negative PRRSV-2 extraction control sample.

### 2.5. Sample Size Calculation and Data Analysis

The sample size was calculated using G Power (v.3.1.9.7, University of Düsseldorf, Düsseldorf, NRW, Germany) [26]. For Studies 1 and 2, a sample size of 12 aliquots as replicates per treatment group was defined, as it provides >80% power to detect a 0.6 difference in RT-qPCR Ct values (assuming a standard deviation of 1.0) between the freeze–thaw treatment (baseline for comparison) and other groups, assuming a type 1 error rate of 5% and dependency between sample means. For Study 3, a sample size of 6 aliquots as replicates per treatment group was defined to detect a 0.6 difference (assuming a standard deviation of 0.7) in Ct values with similar statistical assumptions.

Data analyses were conducted using SAS (v.9.4, SAS Institute, Cary, NC, USA) [27]. For all analyses, a *p*-value < 0.05 was used to determine the statistical significance between the treatments. Each treatment (fresh and freeze–thaw in Study 1 and the 15 treatment combinations in Studies 2 and 3) was considered the experimental unit, while each replicate within the treatments served as the observational unit. Study 2 had double the number of observations compared to 3 as it was tested in duplicate PCR plates.

Study 1: A general linear model (PROC GLM) was used to assess if the PRRSV Ct values on both treatments (fresh and freeze–thaw) were statistically different.

Study 2: A general linear model (PROC GLM) was used to assess whether the two PCR plates’ Ct values differed statistically. Thereafter, a mixed-effects model (PROC MIXED) was used to assess the effect of time and temperature on the Ct values distribution while accounting for random effects attributed to the different plates (1 and 2), i.e., duplicates tested in different extractions and amplification plates. Least square means were computed using Tukey–Kramer adjustment to compare the effects of time and temperatures on Ct values across the study. Freeze–thaw treatment (−20 °C at hour 0) was used as the reference group for these comparisons.

Study 3: A general linear model (PROC GLM) was used to assess the effect of time and temperature on Ct values distribution, followed by the least square means, similar to Study 2.

Model assumptions of normality and linearity were confirmed through Q-Q and residual plots, which showed an acceptable distribution of residuals.

## 3. Results

All samples from Studies 1, 2, and 3 were PRRSV and ISC-positive, with the PRRSV mean Ct values ranging from 18.3 to 19.4 in Study 1, 17.9 to 24.1 in Study 2, and 27.2 to 30.2 in Study 3.

### 3.1. Study 1

The PCR assay’s PRRSV Ct values were obtained for fresh and freeze–thaw treatments. The fresh treatment Ct value mean was 18.3 (95% confidence interval [CI]: 18.2, 18.4), and the freeze–thaw treatment was 19.4 (95% CI: 19.1, 19.6), with a difference of 1.1 (95% CI: 0.9, 1.2), both statistically significantly different (*p* < 0.0001).

### 3.2. Study 2

The stability of PRRSV RNA in a high viral load TF sample by time and temperature was evaluated (Table 1). Each time and temperature included replicates tested in duplicates (*n* = 12), with time 0 for freeze–thaw treatment as the baseline for comparison, with a mean Ct value of 18.0 (95% CI: 17.6, 18.5). There was no statistical difference between the duplicate plates (*p* = 0.13).

**Figure 2:**
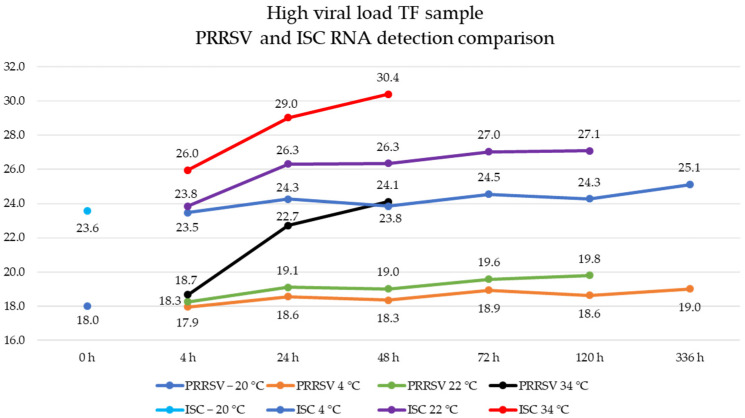
Porcine reproductive and respiratory syndrome virus (PRRSV) and endogenous internal sample control (ISC) RNA detection in high viral load tongue fluid samples stored at different time and temperature points represented by distinct colors. The *Y*-axis represents the cycle threshold values, and the *X*-axis represents the assessed time intervals (hours).

The samples stored at 4 °C were stable from 4 to 336 h, with a Ct value variation of 1 across the evaluated time points. Specifically, there was no statistical difference at 4 °C for 4 h (*p* = 1) and up to 48 h (*p* = 0.72), and a trend toward no difference for 24 h (*p* = 0.049). In contrast, samples stored at 22 °C and 34 °C had higher Ct values than baseline, whereas, after 24 h at 22 °C, all time points were statistically different (*p* < 0.0001), and at 34 °C, all time points were statistically different (*p* < 0.01). Maximum mean Ct values were 19.8 at 22 °C and 24.1 at 34 °C (Figure 2).

For the ISC RNA detection, the baseline sample’s mean Ct value was 23.6 (95% CI: 22.8, 24.3). Samples stored at 4 °C had stable results from 4 to 336 h, with a mean Ct value range of 23.5 to 25.1 across the evaluated time points. Except for treatment stored at 4 °C for 72 h (*p* < 0.001) and 336 h (*p* = 0.0001), all time points were not statistically different from freeze–thaw treatment (*p* ≥ 0.06). Samples stored at higher temperatures had higher Ct values: after 24 h at 22 °C and all time points at 34 °C, samples were statistically different (*p* < 0.0001), with a mean Ct value range across evaluated time points of 23.8 to 27.1 at 22 °C and a range of 26.0 to 30.4 at 34 °C (Figure 2).

### 3.3. Study 3

The stability of PRRSV RNA in a low viral load TF sample by time and temperature was evaluated (Table 2). Each time and temperature included six replicated tests, with time 0 for freeze–thaw treatment as the baseline for comparison, with a Ct value of 27.2 (95% CI: 26.9, 27.4).

**Figure 3:**
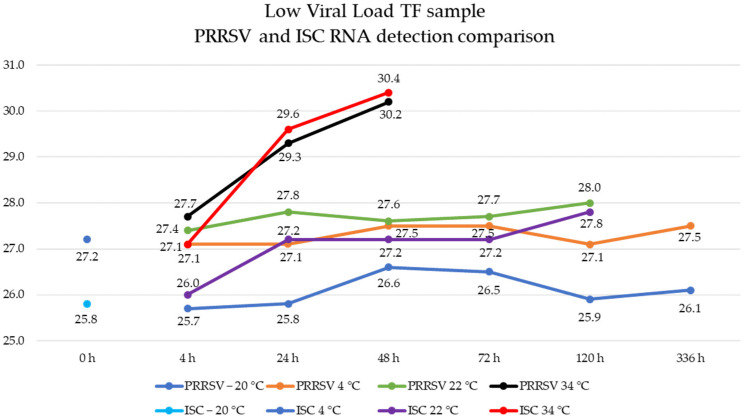
Porcine reproductive and respiratory syndrome virus (PRRSV) and endogenous internal sample control (ISC) RNA detection in low viral load tongue fluid samples stored at different time and temperature points represented by distinct colors. The *Y*-axis represents the cycle threshold values, and the *X*-axis represents the assessed time intervals (hours).

Samples stored at 4 °C were highly stable from 4 to 336 h, with a Ct value variation of 0.3 and no statistical difference (*p* ≥ 0.76) across all time points. In contrast, samples stored at 22 °C and 34 °C had higher Ct values than baseline values. A statistically significant difference was observed at 22 °C stored for 24 h (*p* = 0.02) and for 120 h (*p* = 0.0006), and at 34 °C stored for 24 and 48 h (*p* < 0.0001). Maximum mean Ct values were 28.0 at 22 °C, and 30.2 at 34 °C (Figure 3).

For the ISC RNA detection, the baseline sample’s mean Ct value was 25.8 (95% CI: 25.4, 26.3). Samples stored at 4 °C had stable results from 4 to 336 h, with a mean Ct value range of 25.7 to 26.6 across the evaluated time points. Except for treatment stored at 4 °C for 48 h (*p* = 0.042), all time points were not statistically different from freeze–thaw treatment (*p* ≥ 0.06). Samples stored at higher temperatures had higher Ct values: the storage at 22 °C had a mean Ct value range of 26.0 to 27.8, and the storage at 34 °C had a Ct value range of 27.1 to 30.4 (Figure 3).

## 4. Discussion

Tongue fluids are a population-based sample type that can be used in PRRSV monitoring programs to assess PRRSV circulation, with great advantages, such as being animal welfare-friendly as no live animals are sampled and providing a risk-based approach, as they come from dead animals [7]. However, concerns arise regarding its composition. It is not a sterile sample, and saliva contains enzymes, which could potentially degrade viral RNA [16,17,18]. Suboptimal sampling and storage conditions could compromise the diagnostic quality of the RNA extraction, resulting in a reduced rate of PRRSV detection among infected animals, especially when qPCR Ct values are close to the cut-off [22,24,28,29,30,31]. Thus, understanding the best sample handling and storage conditions can improve TF PRRSV RNA RT-qPCR detection rates.

In Study 1, although the fresh treatment had statistically lower Ct values of up to 1.1 than the freeze–thaw treatment, this variation is considered acceptable from a molecular diagnostic standpoint as it represents an approximately 2-fold difference in the quantity of the detected target. Moreover, the freeze–thaw treatment in Study 2 had a mean Ct value of 18.0, 0.3 Ct value lower than the fresh sample in Study 1. As these aliquots originated from the same initial TF, this variation could be explained by pipetting, extraction and amplification process, or even test efficiency [30].

Notably, in Studies 2 and 3, PRRSV RNA in TF was stable at 4 °C for up to 14 days (e.g., 336 h), similar to processing fluids [29]. Likewise, oral fluids were reported as stable at −20 °C, 4 °C and 10 °C for up to 12 days, whereas when stored at 20 °C or 30 °C, the RNA detection declined significantly within 12 h [31]. There are greater concerns regarding high Ct value TF, i.e., low viral load, as detection may be compromised due to the closeness of the threshold cutoff and due to more variable Ct results when approaching the last PCR cycles, as the confidence interval of the target copy number is larger when the initial target copy number is low [30].

In this study, the dead animals were kept at room temperature (22 °C) before the sampling time point, which was one of the temperatures evaluated in this study, with a variability ≤3.3 ISC Ct value. Based on the study’s findings, although producers and veterinarians would not normally collect samples from animals found dead after several days due to good sampling practices, the ISC was still viable in the sample for up to 5 days (e.g., 120 h) at 22 °C. Lastly, ISC RNA detection remained stable when stored at 4 °C for up to 14 days (e.g., 336 h), with patterns similar to what was observed for PRRSV RNA detection. These consistent ISC detection results support the use of TF in PRRSV monitoring programs. As described elsewhere, failures in detecting ISC provide sufficient justification for conducting additional testing and/or collecting new samples [21].

Although the main idea of the study was to mimic similar practices in the field, there were two limitations: (1) sampling aliquoting process: TF can contain environment particles, such as feces or feed, which may negatively affect handling processes, such as pipetting [10]. To avoid unequal aliquots and to target more homogenized samples, TFs were centrifuged before aliquoting to remove the sediments from the supernatant; (2) all aliquots from studies 2 and 3 were frozen to allow the testing occurrence on the same day. Considering the freeze–thaw process could potentially impact RNA detection, samples used in this study were thawed at 4 °C to reduce this effect [24].

## 5. Conclusions

In conclusion, TF PRRSV RNA detection is highly stable at ≤4 °C for up to 14 days, with a Ct value increase of ≤1. However, its stability decreases with higher temperatures and longer storage periods, such as 22 °C and 34 °C up to five days, similar to processing fluids and oral fluids. Therefore, producers and veterinarians are encouraged to store TF at ≤4 °C for no more than 14 days before molecular testing, with cold storage maintained during the transport, ensuring optimal diagnostic quality for PRRSV RNA detection by RT-qPCR. In situations of uncertainty regarding storage conditions, such as transport delay, the PCR results can be negatively affected, and each particular case must be reviewed between veterinarians and diagnosticians.

## Figures and Tables

**Table 1:** Time and temperature effects on the detection of porcine reproductive and respiratory syndrome virus RNA in a high viral load tongue fluid by RT-qPCR, with, respectively, mean Ct values (95% confidence interval) based on six replicates per treatment.

Time in Hours	−20 °C	4 °C	22 °C	34 °C
0	18.0 (17.6, 18.5)			
4		17.9 (17.5, 18.4) *p*-value = 1.0	18.3 (17.8, 18.7) *p*-value = 0.9670	18.7 (18.2, 19.1) * p-value = 0.0038
24		18.6 (18.1, 19.0) * p-value = 0.0492	19.1 (18.7, 19.5) * p-value < 0.0001	22.7 (22.3, 23.2) * p-value < 0.0001
48		18.3 (17.9, 18.8) *p*-value = 0.7293	19.0 (18.6, 19.5) * p-value < 0.0001	24.1 (23.7, 24.6) * p-value < 0.0001
72		18.9 (18.5, 19.4) * p-value < 0.0001	19.6 (19.1, 20.0) * p-value < 0.0001	
120		18.6 (18.2, 19.1) * p-value = 0.0098	19.8 (19.4, 20.2) * p-value < 0.0001	
336		19.0 (18.6, 19.5) * p-value < 0.0001		

Blank cells indicate that the condition was not assessed. * Statistically different from freeze–thaw treatment, i.e., −20 °C at hour 0.

**Table 2:** Time and temperature effects on the detection of porcine reproductive and respiratory syndrome virus RNA in a low viral load tongue fluid by RT-qPCR, with, respectively, mean Ct values (95% confidence interval) based on six replicates per treatment.

Time in Hours	−20 °C	4 °C	22 °C	34 °C
0	27.2 (26.9, 27.4)			
4		27.1 (26.9, 27.4) *p*-value = 1.0	27.4 (27.1, 27.6) *p*-value = 0.9989	27.7 (27.4, 28.9) *p*-value = 0.3131
24		27.1 (26.8, 27.3) *p*-value = 1.0	27.8 (27.6, 28.1) * p-value = 0.0274	29.3 (29.0, 29.5) * p-value < 0.0001
48		27.5 (27.3, 27.8) *p*-value = 0.7634	27.6 (27.3, 27.8) *p*-value = 0.6224	30.2 (30.0, 30.5) * p-value < 0.0001
72		27.5 (27.3, 27.8) *p*-value = 0.8384	27.7 (27.4, 27.9) *p*-value = 0.2961	
120		27.1 (26.8, 27.3) *p*-value = 1.0	28.0 (27.8, 28.3) * p-value = 0.0006	
336		27.5 (27.3, 27.8) *p*-value = 0.8059		

Blank cells indicate that the condition was not assessed. * Statistically different from freeze–thaw treatment, i.e., −20 °C at hour 0.

## Data Availability

The raw data supporting the conclusions of this article will be made available by the authors on request.
